# Perturbation-Based Balance Training in Postoperative Individuals With Degenerative Cervical Myelopathy

**DOI:** 10.3389/fbioe.2020.00108

**Published:** 2020-02-20

**Authors:** Yi-Shan Cheng, Andy Chien, Dar-Ming Lai, Ya-Yun Lee, Chih-Hsiu Cheng, Shwu-Fen Wang, Ya-Ju Chang, Jaw-Lin Wang, Wei-Li Hsu

**Affiliations:** ^1^School and Graduate Institute of Physical Therapy, College of Medicine, National Taiwan University, Taipei, Taiwan; ^2^Division of Physical Therapy, Department of Physical Medicine and Rehabilitation, Shin Kong Wu Ho-Su Memorial Hospital, Taipei, Taiwan; ^3^Department of Physical Therapy, Graduate Institute of Rehabilitation Science, China Medical University, Taichung, Taiwan; ^4^Department of Surgery, National Taiwan University Hospital, Taipei, Taiwan; ^5^Physical Therapy Center, National Taiwan University Hospital, Taipei, Taiwan; ^6^School of Physical Therapy and Graduate Institute of Rehabilitation Science, College of Medicine, Chang Gung University, Taoyuan, Taiwan; ^7^Department of Biomedical Engineering, College of Medicine and College of Engineering, National Taiwan University, Taipei, Taiwan

**Keywords:** degenerative cervical myelopathy, decompression surgery, perturbation-based balance training, postural control, rehabilitation

## Abstract

Degenerative cervical myelopathy (DCM) is a common aging condition caused by spinal cord compression. Individuals with DCM often presented with residual balance and functional impairments postoperatively. Perturbation-based balance training (PBT) has been shown to have positive effects on populations with neurological disorders but has yet to be investigated in DCM. The objective of this study was therefore to evaluate the effects of PBT on balance and functional performance in postoperative individuals with DCM. Fifteen postoperative individuals with DCM (DCM group) and 14 healthy adults (healthy control group) were recruited. The DCM group received a 4-weeks PBT using a perturbation treadmill. The outcome measures included mean velocity of center of pressure (COP) during quiet standing; center of mass (COM) variance and reaction time to balance perturbation during standing with forward and backward perturbation; gait speed during level ground walking; Timed Up and Go Test (TUG) and disability questionnaire scores including Visual Analog Scale, Neck Disability Index, and Lower Extremity Function of Japanese Orthopaedic Association Cervical Myelopathy Evaluation Questionnaire. The assessments were conducted pre- and post-training postoperatively for the DCM group but only once for the healthy control group. Significant improvements were observed in the mean velocity of COP, COM variance, reaction time, gait speed, and TUG in the DCM group. Disability questionnaire scores were not significantly different after training in DCM group. For between-group comparisons, significant differences that were observed pre-training were not observed post-training. The 4-weeks PBT is a potential rehabilitation strategy for addressing balance and functional impairment in postoperative individuals with DCM. In addition, the post-training performance in the DCM group exhibited trends comparable to those of age-matched healthy controls. Furthermore, the training regimens offer a practical reference for future studies on populations with balance disorders. Future studies complemented with neurophysiological assessments could reveal more information of the underlying mechanisms of PBT.

## Introduction

Degenerative cervical myelopathy (DCM) is a common aging condition caused by spinal cord compression (Gibson et al., 2018). In North America, the incidence of DCM was estimated to be 605 individuals per million (Nouri et al., 2015). As an overarching term, DCM encompasses cervical spondylotic myelopathy, ossification of perispinal ligaments, and intervertebral disk disease (Nouri et al., 2015). Ischemia or necrosis of the compressed spinal cord can lead to the manifestation of neurological symptoms, such as paresthesia, weakness, incontinence, spasticity, and pain (Kato and Fehlings, 2016). Furthermore, these neurological deficits may contribute to balance instability and functional limitations (Tracy and Bartleson, 2010). Cervical decompression surgery is a common intervention for individuals with DCM when conservative management failed to achieve adequate symptom relief (Kato and Fehlings, 2016; Rhee et al., 2017).

Over the last decades, the number of DCM-related surgeries has increased, which has been attributed to advancement in medical technologies and diagnostic precision (Passias et al., 2017). Surgery is aimed at expanding the spinal canal to re-establish neuronal conduction and vascular circulation, as well as stabilizing hypermobile segments to prevent further spinal deformity (Tracy and Bartleson, 2010). Although positive clinical recovery is commonly reported after surgery (Fehlings et al., 2018; Kopjar et al., 2018), some individuals still present with persistent neurological symptoms due to severely or prolonged compression of the spinal cord (Sampath et al., 2000; Tetreault et al., 2015). In addition, despite the repair of neuronal integrity after surgery, pathological changes in the spinal cord may still alter the motor control scheme to compensate for sensorimotor dysfunction (Lin et al., 2019).

Several studies have reported balance instability in individuals with spinal disease (Boudreau and Falla, 2014; Hsu et al., 2014, 2019; Wong et al., 2019). Compared with healthy controls, a greater postural sway with increased muscle activation in the trunk and lower extremities was observed during quiet standing in individuals with DCM (Yoshikawa et al., 2008; Haddas et al., 2018b). In addition, delayed neuromuscular recruitment and postural unsteadiness under perturbation were reported in individuals with DCM (Nardone et al., 2008). Although an improvement in balance have been reported after surgery (Tanishima et al., 2017), whether balance deficits associated with DCM can be fully reversed postoperatively remain inconclusive due to the lack of comparison with healthy adults.

Functional impairment is another key issue in individuals with DCM. Approximately 35–45% of individuals with DCM have ambulatory disability, and 35–39% of which still suffer from gait disturbances after medical or surgical intervention (Sampath et al., 2000). Although the improved ankle power during walking was reported in postoperative individuals with DCM (Malone et al., 2015), inconsistent results were found with regard to spatiotemporal and kinematic gait parameters following surgery (Malone et al., 2015; Haddas et al., 2018a). The improvement of disability status, such as the Neck Disability Index (Fehlings et al., 2013) and modified Japanese Orthopaedic Association score (Furlan et al., 2011; Zika et al., 2018), was also suggested after surgery. Nevertheless, functional recovery based on walking and balance ability may reach a plateau 6 months after surgery (Furlan et al., 2011), which may represent the natural recovery process of the impaired spinal cord. In addition, the recovery plateau highlights the need for a rehabilitation program for postoperative individuals with DCM to address residual impairments observed after surgery.

**FIGURE 1:**
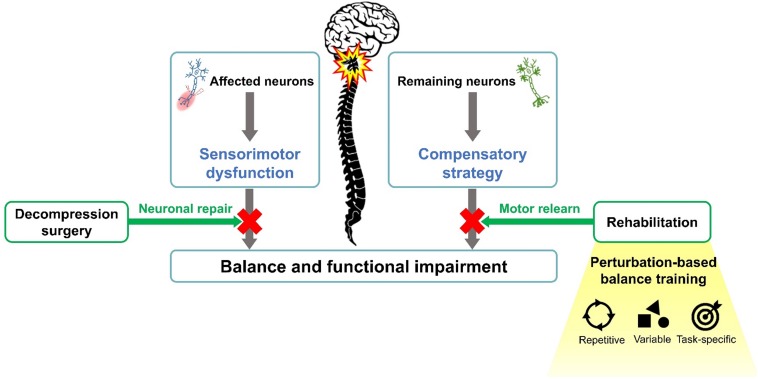
Mechanism of perturbation-based balance training.

Motor relearning is an essential concept in neurorehabilitation (Edgerton et al., 2001; Mulder and Hochstenbach, 2001; Kitago and Krakauer, 2013). In individuals with spinal cord impairment, remaining synapses in neuronal tracts can regenerate due to the plasticity, and the neuron circuitry can relearn motor skills if proper reinforcements were offered (Edgerton et al., 2001; Mulder and Hochstenbach, 2001; Kakebeeke et al., 2006; Kitago and Krakauer, 2013). Therefore, repetitive practice in a task-specific environment can facilitate the reacquisition of a motor skill (Edgerton et al., 2001; Mulder and Hochstenbach, 2001; Kakebeeke et al., 2006). Based on such concepts, perturbation-based balance training (PBT) is one of the clinical applications of task-specific balance rehabilitation (Figure 1). When exposed to repeated perturbations, the neuromotor system would be stimulated to develop necessary neurophysiological changes and sensorimotor skills to prevent falling (Grabiner et al., 2014). PBT has been reported to be potentially effective for populations with balance disorders, including enhanced postural stability (Chien and Hsu, 2018; Handelzalts et al., 2019; Klamroth et al., 2019), decreased fall incidence (Gerards et al., 2017; Mansfield et al., 2017), and improved functional performance (Steib et al., 2017) in individuals with neurological conditions after training.

Therefore, this study aimed to investigate the effects of PBT on balance and functional performance in postoperative individuals with DCM. We hypothesized that PBT can enhance balance and functional performance in postoperative individuals with DCM. Furthermore, the performance after training can exhibit trends comparable to those of age-matched healthy controls.

## Materials and Methods

### Study Procedure

**FIGURE 2:**
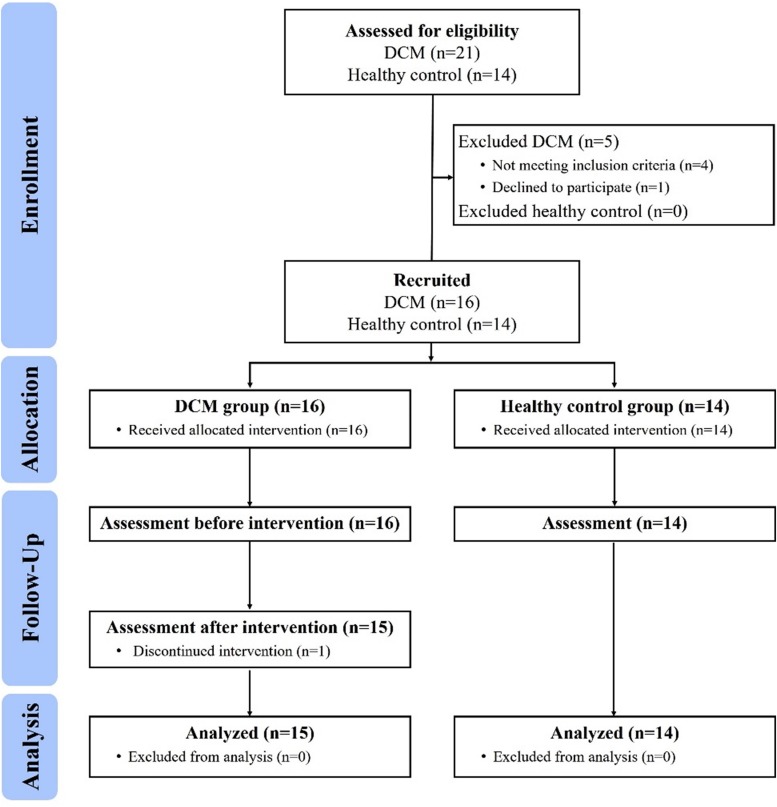
Flow chart of the study.

A 2-arm prospective controlled clinical trial was conducted (Figure 2). Participants were recruited from the neurosurgical clinic at National Taiwan University Hospital (NTUH). After explanation of the study procedure, all participants provided written informed consent and were allocated as the DCM group or healthy control group. For the DCM group, assessments were conducted within 1-week before (pre-training) and after (post-training) the intervention. In the healthy control group, assessments were only conducted once. The study was approved by the Research Ethics Committee of NTUH (Reference number: 201512167RINB) and registered at ClinicalTrials.gov (Registration number: NCT02842775).

### Participants

For the DCM group, inclusion criteria were (1) aged between 40 and 80 years, (2) underwent cervical decompression surgery with a diagnosis of cervical myelopathy based on relevant image examinations, (3) able to walk independently for ≥10 m, and (4) able to stand upright ≥30 s. Exclusion criteria were with a history of (1) traumatic spinal cord injury, (2) pathological musculoskeletal disorders, (3) neurological impairment, and (4) other balance disorders such as vestibular dysfunction. For the healthy control group, inclusion criteria were (1) aged between 40 and 80 years, (2) no neck pain experienced in the last 6 months, (3) able to walk independently for ≥10 m, and (4) able to stand upright for ≥30 s. Exclusion criteria for the healthy control group were similar to those for the DCM group.

### Intervention

**FIGURE 3:**
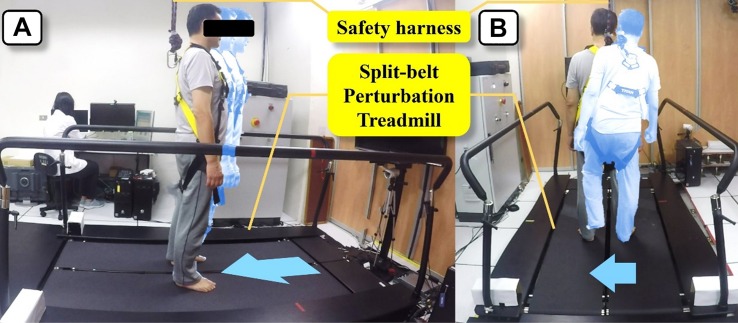
Perturbation-based balance training using a split-belt perturbation treadmill. **(A)** Training during standing. **(B)** Training during walking.

The intervention and assessments was delivered at Movement Science Lab of the School and Graduate Institute of Physical Therapy in National Taiwan University. Participants in the DCM group received a 4-weeks PBT program using a split-belt perturbation treadmill (QQ-mill, Motekforce Link, Amsterdam, Netherlands) (Figure 3). For participant safety, a suspension harness attached to the ceiling was employed, and the entire session was conducted under the supervision of a certified physical therapist.

Two 1-h training sessions were conducted weekly and eight sessions in total per participant. Each session includes 20 forward, 20 backward, and 40 lateral perturbations (20 right-to-left and 20 left-to-right) (Chien and Hsu, 2018). Unexpected perturbations were triggered during standing and walking. For standing training, perturbation speeds were set at 0.15–0.20 m/s in the forward direction, 0.20–0.25 m/s in the backward direction, and 0.09–0.18 m/s in the lateral direction. For walking training, the treadmill speed was set at 0.8 comfortable walking speed determined by the 10-m walking test before training with an increment of 0.05 m/s for each session. Perturbation speeds were customized based on the treadmill speed of the prevailing session (forward perturbation: treadmill speed −0.4 m/s; backward perturbation: treadmill speed +0.50 m/s; and lateral perturbation: 0.09–0.18 m/s).

### Data Collection and Process

Kinematic data were collected using a VICON motion analysis system with 10 infrared cameras (VICON Bonita, Oxford Metrics Ltd., Oxford, United Kingdom). Forty-five 14-mm spherical reflective markers were attached to anatomical landmarks according to a Plug-In-Gait marker set with a 15-segment model to calculate the whole-body center of mass (COM). Kinetic data were collected using three force platforms (model OR6-7, AMTI, MA, United States) embedded on the 5-m walkway. Muscle activation data were collected using surface wireless electromyographic (EMG) electrodes (Trigno, Delsys, Inc., Boston, MA, United States). VICON Nexus Plug-In-Gait biomechanical modeling software and a program custom-written in MATLAB (The MathWorks, Inc., Natick, MA, United States) were used to process and analyze kinematic, kinetic, and EMG data. In addition, EMG data were filtered using a bi-directional second-order Butterworth digital filter for eliminating artificial phase shifts induced during filtering.

### Outcome Measures

Static postural control was assessed using mean velocity of center of pressure (COP) during quiet standing with eyes closed. Participants were asked to stand with feet shoulder-width apart on a force platform with eyes closed for 40 s. The mean velocity of COP was calculated as the average velocity of COP data (Prieto et al., 1996).

**FIGURE 4:**
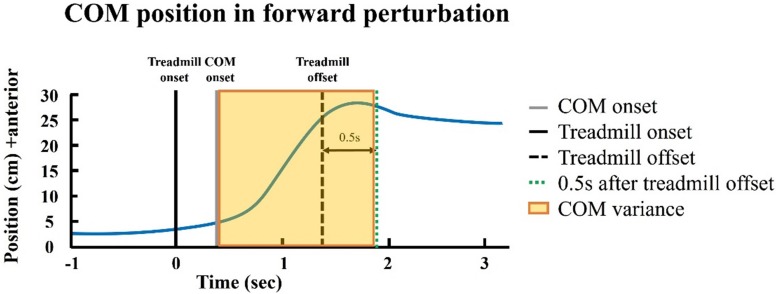
The center of mass (COM) variance in the forward perturbation.

Dynamic postural control was assessed using COM variance and reaction time during standing with perturbation. Participants were asked to stand as still as possible on the perturbation treadmill without holding on to the rail. Based on the average duration of active reaction to perturbation (Nataraj et al., 2012), the COM variance was defined as the variance of the COM position from the COM onset to the treadmill offset +0.5 s (Figure 4). The reaction time was defined from the treadmill onset to the muscle activation onset. The onset and offset events of COM and treadmill movement were defined based on the COM and treadmill velocity (Hsu et al., 2013). The onset of the muscle activation was defined as mean +3 times of SD of the 1 s of baseline value of EMG signals. The target muscles were tibialis anterior and gastrocnemius for the forward and backward perturbations. The speeds of perturbations were set at 0.15 m/s for forward perturbation and 0.2 m/s for backward perturbation.

Functional performance was assessed using gait speed and the Timed Up and Go Test (TUG) (Shumway-Cook et al., 2000). In level-ground walking, participants walked barefoot along a 5-m walkway with their comfortable speed, and the gait speed was calculated.

Disability questionnaires included the Visual Analog Scale (VAS) (Jensen et al., 2003; Alghadir et al., 2018), Neck Disability Index (NDI) and Lower Extremity Function of Japanese Orthopaedic Association Cervical Myelopathy Evaluation Questionnaire (JOACMEQ-LEF). The NDI is an instrument used to assess impairment of daily activity due to neck discomfort (Vernon and Mior, 1991). The higher the percentage, the greater impairment of daily activity. JOACMEC is an instrument for assessing the neurological function (Fukui et al., 2007). It consists of five domains including functions of cervical spine, upper extremity, lower extremity, bladder, and quality of life. A high score indicates a favorable condition. The Chinese version (Chien et al., 2014) and the lower extremity function were applied in this study.

### Statistical Analysis

Statistical analysis was conducted with PASW Statistics 18.0 for Windows (SPSS, Chicago, IL, United States). Baseline values between groups were compared using the Mann–Whitney *U* test for continuous parameters and using the Chi-square test for categorical parameters. Within-group comparison were conducted using the Wilcoxon signed-rank test. Between-group comparison were conducted using the Mann–Whitney *U* test. The alpha level was set at 0.05. Within-group effect sizes (ESs) were calculated using Cohen’s *d*. The *d* value between 0.20 and 0.49 indicated a small effect; a value between 0.50 and 0.79 indicated a moderate effect; a value >0.79 was considered a large effect. The sample size was determined from a preliminary study.

## Results

A total of 15 postoperative individuals with DCM and 14 healthy adults were included in this study. The demographic data are listed in Table 1. No significant differences were observed for sex, age, height, weight, and body mass index between groups.

**TABLE 1:** Demographic data.

	**DCM group, *n* = 15 (mean ± SD)**	**Healthy control group, *n* = 14 (mean ± SD)**	***p*-value**
Sex (*n*, male/female)	10/5	5/9	0.14
Age (years)	64.0 ± 5.3	67.4 ± 5.9	0.08
Height (cm)	163.8 ± 6.2	159.7 ± 5.1	0.07
Weight (kg)	67.4 ± 9.4	61.0 ± 6.3	0.08
BMI (kg/m^2^)	25.1 ± 3.0	24.0 ± 2.8	0.33
Symptoms duration (months)	66.3 ± 96.8	–	–
Duration after surgery (months)	16.7 ± 14.3	–	–
Surgical method (anterior/posterior)	10/5	–	–
VAS (0–10)	2.4 ± 2.3	–	–
NDI (%)	12.7 ± 8.3	–	–
JOACMEQ-LEF (%)	74.3 ± 21.8	–	–

*BMI, Body mass index; VAS, Visual Analog Scale; NDI, Neck Disability Index; JOACMEQ-LEF, Lower Extremity Function of Japanese Orthopaedic Association Cervical Myelopathy Evaluation Questionnaire.*

### Static Postural Control

**FIGURE 5:**
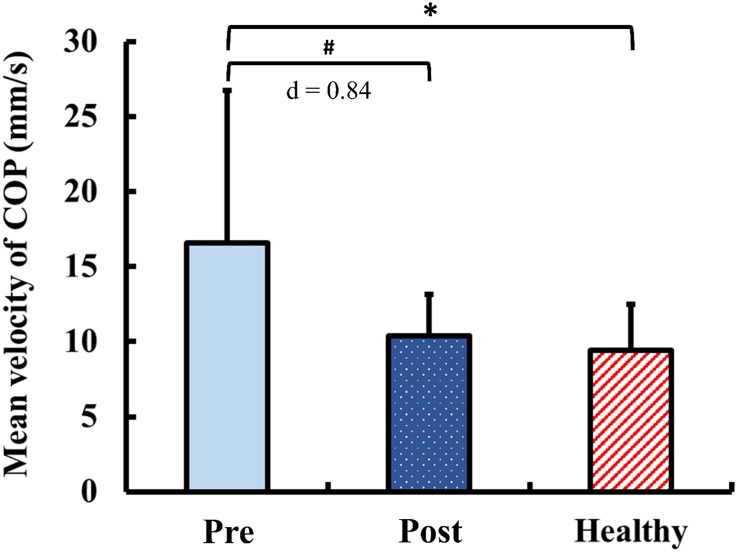
Results for static postural control: mean velocity of center of pressure (COP) during quiet standing with eyes closed. The symbol “^∗^” indicated a significant difference between the DCM group and the healthy control group: *p* < 0.05. The symbol “#” Indicated a significant difference in the DCM group before and after training: *p* < 0.05.

The results of static postural control obtained during quiet standing with eyes closed are presented in Figure 5. Pre-training, the mean velocity of COP in the DCM group was significantly greater than that of the healthy control group (*p* < 0.01). Post-training, the mean velocity of COP in the DCM group significantly improved from the pre-training value with large ES (*p* = 0.02, *d* = 0.84) and there was no significant between-group difference of the mean velocity of COP after training (*p* = 0.34).

### Dynamic Postural Control

**FIGURE 6:**
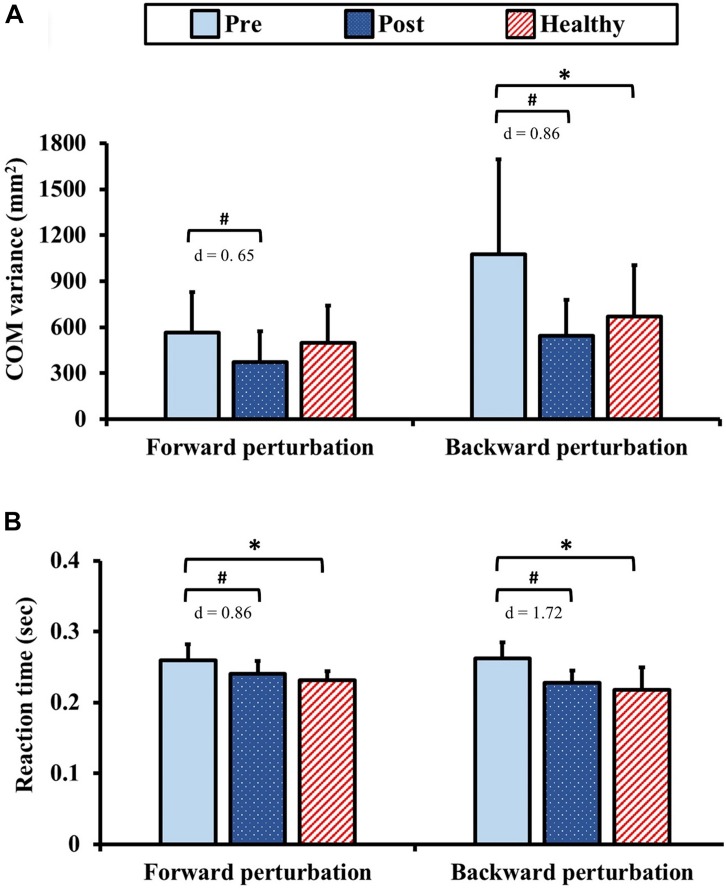
Results for dynamic postural control: **(A)** center of mass (COM) variance and **(B)** reaction time during standing with perturbation. The symbol “^∗^” indicated a significant difference between the DCM group and the healthy control group: *p* < 0.05. The symbol “#” indicated a significant difference in the DCM group before and after training: *p* < 0.05.

The results of dynamic postural control during standing with perturbation are presented in Figure 6. Pre-training, the COM variance exhibited no significant between-group difference (*p* = 1.00) for the forward perturbation. Post-training in the forward perturbation, the COM variance in the DCM group significantly improved from the pre-training value with moderate ES (*p* = 0.03, *d* = 0.65). In addition, there was no significant between-group difference of the COM variance in the forward perturbation after training (*p* = 0.11). In contrast, the COM variance in the DCM group was significantly greater than that of the healthy control group (*p* = 0.04) for the backward perturbation pre-training. Post-training, the COM variance in the DCM group significantly improved from the pre-training value with large ES (*p* < 0.01, *d* = 0.86) and there was no significant between-group difference of the COM variance in the backward perturbation after training (*p* = 0.34).

In terms of the reaction times, the reaction times in the DCM group were significantly longer than those of the healthy control group pre-training (forward and backward: *ps* < 0.01). Post-training, the reaction times in the DCM group significantly improved from the pre-training values with large ES (forward: *p* < 0.01, *d* = 0.86; backward: *p* < 0.01, *d* = 1.72) and no significant between-group differences were identified (forward: *p* = 0.20, backward: *p* = 0.34).

### Functional Performance

**FIGURE 7:**
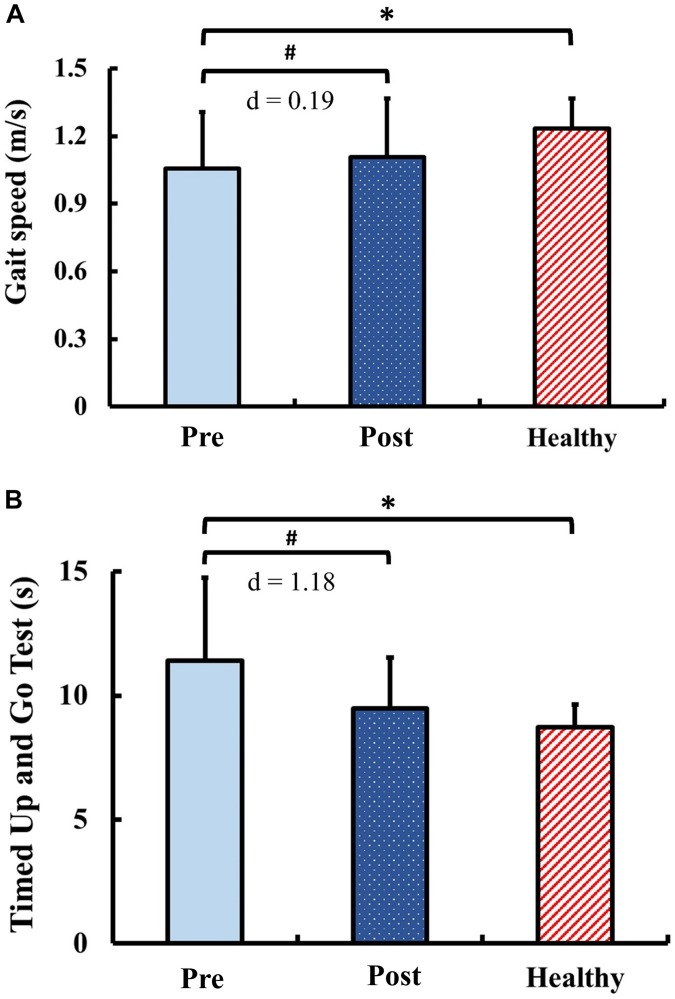
Results for functional performance: **(A)** gait speed and **(B)** Timed Up and Go Test. The symbol “^∗^” indicated a significant difference between the DCM group and the healthy control group: *p* < 0.05. The symbol “#” indicated a significant difference in the DCM group before and after training: *p* < 0.05.

The results of functional performance are presented in Figure 7. Pre-training, the gait speed and the TUG in the DCM group were significantly slower than those of the healthy control group (gait speed: *p* = 0.03; TUG: *p* < 0.01). Post-training, the gait speed and the TUG in the DCM group significantly improved from the pre-training values with marginal small ES (gait speed: *p* = 0.04; *d* = 0.19) and with large ES (TUG: *p* < 0.01; *d* = 1.18). There were no significant between-group differences of the gait speed and the TUG after training (gait speed: *p* = 0.15; TUG: *p* = 0.15).

### Disability Questionnaires

In the DCM group, no significant changes of the questionnaire scores were observed after training, including VAS (*p* = 1.00), NDI (*p* = 0.30), and JOACMEQ-LEF (*p* = 0.05).

## Discussion

The present study aimed to investigate the effects of a 4-week PBT on balance and functional performance in postoperative individuals with DCM. The findings supported our hypothesis that PBT can improve balance and functional performance in postoperative individuals with DCM, and the performance after training exhibited trends comparable to those of age-matched healthy controls.

### Residual Balance Instability and Functional Impairment After Surgery

The existence of poor balance and functional performance in individuals with DCM before surgical intervention has been reported by several studies (Nardone et al., 2008; Yoshikawa et al., 2008; Haddas et al., 2018b). Our study further demonstrated residual balance instability and functional impairments in postoperative individuals with DCM.

For postural control assessments, the observed inferior performance compared with healthy controls suggested impaired proprioceptive sensation and poor neuromuscular control in postoperative individuals with DCM. Chronic compression of spinal cord can result in permanent viscoelastic and histological damage to the neuronal cells (Carlson et al., 2003). Subsequently, sensorimotor dysfunction could be caused by the disruption of neuronal transmission. Even when individuals with DCM undergo decompression surgery, such permanent damage would still result in persistent sensorimotor dysfunction (Goncalves et al., 2016). In addition, adaptive motor strategies can be further developed to compensate for the sensorimotor dysfunction under the guidance of impaired proprioceptive sensation inputs (Lin et al., 2019).

Notably, the significant between-group difference of COM variance during perturbation assessment was observed only during the backward perturbation but not the forward perturbation. It is speculated that unexpected forward perturbation is a challenging task even for healthy elderly (Wang et al., 2017) thus contributing to the lack of between group differences. Nevertheless, the results indicated that since tripping is one of the leading cause of falls in elderly (Stolze et al., 2004), simulating tripping scenarios such as backward perturbation could serve as a sensitive tool to differentiate the balance ability between individuals with neurological disorders.

The significant functional impairments of gait speed and TUG were also observed in individuals with DCM postoperatively in the current study. These poor mobility-related performance might be explained by the alterations of neuromuscular control during walking, such as reduced ankle plantar flexion at toe off (Lee et al., 2011) and prolonged activation duration of tibialis anterior (Malone et al., 2015). In addition, the aforementioned inadequate proprioception and neuromuscular control observed in the postural control assessments may also further contribute to the poor functional performance.

### Improvement of Balance and Functional Performance After Training

Our study demonstrated that PBT could improve static postural control in postoperative individuals with DCM. The decreased mean velocity of COP after training indicated the reduced postural fluctuation during standing (Masani et al., 2014), which implied the enhanced static balance performance and the efficient neuromotor control strategy. Several tasks have been employed in previous studies to assess the effects of PBT on static postural control, including sitting, quiet standing, and Romberg stance (Chien and Hsu, 2018; Handelzalts et al., 2019). In the current study, we employed the stance task without visual inputs because the proprioceptive system plays a major role in maintaining stability during such task (Horak, 2006) and the enhanced performance in such a task can infer improvement in proprioceptive sensation after training. However, the improvements observed in the patient population in the present study was not found in the elderly population based on our former study (Chien and Hsu, 2018). Such differences could be attributed to the population characteristics. The inferior static postural control of patients may provide more room for balance improvement compared with healthy elderly. Therefore, individuals with balance disorders may benefit more from PBT when compared with healthy populations.

The improvement of dynamic postural control after PBT was also observed in this study. The decreased COM variance and the reaction time in response to perturbation indicated the enhanced reactive balance performance (Handelzalts et al., 2019). With repetitive exposure to perturbations, the participants could learn the efficient strategy to maintain stability, and therefore accelerate the re-learning of motor skills to against fall (Grabiner et al., 2014; Handelzalts et al., 2019). Consistent with previous studies, the decreased COM variance (Chien and Hsu, 2018) and muscle latency (Krause et al., 2018) under perturbation suggested improved balance steadiness and neuromuscular recruitment in response to external perturbation. A similar observation was made in subacute individuals with stroke, indicating that PBT could facilitate balance recovery under dynamic conditions (Handelzalts et al., 2019). In elderly population, PBT could also improve proactive and reactive stability when individuals encounter slip-like perturbations during level-ground walking (Liu et al., 2017). Moreover, enhanced proprioception observed in this study could also improve the stability under perturbation.

Functional improvements following PBT were also found in the present study. Optimal effects of PBT on mobility-related dynamic balance in individuals with Parkinson’s disease (Steib et al., 2017) and stroke (Handelzalts et al., 2019) have been reported in previous studies. The positive feedback from treadmill belts may induce the auto correction of gait patterns and increase locomotor pattern generator activity within the central nervous system (Harkema, 2001). Additionally, unexpected perturbations during walking can modulate neuromuscular activation (Steib et al., 2017) and therefore lead to improvements in movement efficiency.

Insignificant changes in questionnaire outcomes following PBT could be resulted from the ceiling effect of such self-assessed instruments. In the present study, the outcomes of biomechanical and physical assessments demonstrated balance and functional impairments in postoperative individuals with DCM. However, the average baseline value of VAS was <3.3, which was the level of acceptable pain in postoperative individuals (Myles et al., 2017). In addition, more than 60% of the participants had a NDI score within the range of mild disability (MacDermid et al., 2009), and one-third of those had a JOACMEQ-LEF score more than 90 points at baseline (Fukui et al., 2009). Items included in the questionnaires may not be adequately sensitive to reflect balance and functional deficits, especially with generic questionnaire that do not necessary focus on the assessment of aforementioned sub-domains. Additionally, the unawareness of their balance instability may lead to the overestimation of their balance capacity when responding to questionnaires. Therefore, objective biomechanical and physical assessments should be included in the evaluation of balance and functional performance.

### Possible Underlying Mechanism of Perturbation-Based Balance Training

The underlying mechanisms of balance training have been discussed extensively (Taube et al., 2007; Sehm et al., 2014; Ruffieux et al., 2018; Helm et al., 2019). Based on the concept of motor relearning, PBT can provide abundant sensory stimulation and therefore induce the storage of motor program to prevent falling (Kakebeeke et al., 2006). Motor skills, with enhanced neuromuscular control and inter-joint coordination, can be facilitated through repetitive perturbation stimulations (McCrum et al., 2017; Krause et al., 2018). In postoperative individuals with DCM, residual balance instability after surgery may probably result from the compensatory motor strategies following sensorimotor dysfunction (Edgerton et al., 2001; Mulder and Hochstenbach, 2001; Kitago and Krakauer, 2013). Therefore, PBT may enhance balance performance through the relearning of appropriate movement patterns to maintain stability (Krause et al., 2018), which further correct ineffective compensatory motor strategies.

Neurophysiological changes within the primary motor cortex and the corticospinal pathway have been presumed to regulate balance control (Taube et al., 2007). Inappropriate corticomotor modulation can be an internal factor leading to balance instability (Baudry et al., 2014). For individuals with DCM, impaired delivery of proprioceptive signals through posterior columns has been observed (Lee et al., 2011). In addition, persistent inferior corticospinal conductivity may lead to delayed body adjustments while attempting to maintain postural stability (Nicotra et al., 2013). Balance training is proposed to regulate the motoneuronal excitability and induce the corticomotor reorganization (Taube et al., 2007). In addition, improved postural control with altered neurophysiological presentation, such as reduced overactivation of the brain in individuals with balance disorders, has been reported after balance training (Sehm et al., 2014; Ruffieux et al., 2018). Therefore, the neurophysiological alterations could also be some of the factors driving the optimized balance performance in postoperative individuals with DCM after training. Future studies complemented with neurophysiological assessments could reveal more information of the underlying mechanisms of PBT.

### Study Limitations

Our study has several limitations. First, due to the basic balance requirements for our assessments, individuals who could not walk or stand upright independently were excluded from the study. Therefore, the conclusions obtained may not be generalized to more severely disabled populations. Second, because part of the assessment is similar to the training program, some scholars may argue that the improvement could be resulted from task familiarity (Handelzalts et al., 2019). However, perturbations were triggered unexpectedly; therefore, responses should be spontaneous but not be based on familiarity. Third, lack of a comparison group receiving only treadmill training may raise concerns on whether the effects of the training were attributable to both PBT and treadmill training (Steib et al., 2017; Handelzalts et al., 2019). However, the more pronounced neuromuscular modulation effects with the addition of perturbation to balance training compared with conventional balance training were reported previously (Freyler et al., 2016; Krause et al., 2018). In addition, the effectiveness of PBT may be challenged without the follow-up assessment for the control group. Therefore, further study with pre- and post-assessments for the control group could provide a more powerful evidence for PBT. Overall, the aforementioned limitations should be taken into account when interpreting the findings of the study.

## Conclusion

The present study provides key evidence of the functional balance deficits in postoperative DCM patients that could be improved with a 4-weeks PBT program. To the best of our knowledge, this is the first study of postoperative balance rehabilitation in individuals with DCM. In addition, PBT can serve as a novel rehabilitation strategy to dispense residual balance and functional impairment after surgery. Furthermore, the training regimens in this study offers a practical reference for future research in other populations.

## Data Availability Statement

The datasets generated for this study are available on request to the corresponding author.

## Ethics Statement

The studies involving human participants were reviewed and approved by the Research Ethics Committee of the National Taiwan University Hospital. The patients/participants provided their written informed consent to participate in this study.

## Author Contributions

All authors formulated the ideas of the present study, discussed the results, and contributed to the final manuscript. W-LH managed the project with the participants provided by D-ML. Y-SC and D-ML carried out the data collection with the facilities and equipment provided by D-ML, Y-YL, and W-LH. Y-SC and W-LH wrote the manuscript with support from AC, D-ML, Y-YL, C-HC, S-FW, Y-JC, and J-LW.

## Conflict of Interest

The authors declare that the research was conducted in the absence of any commercial or financial relationships that could be construed as a potential conflict of interest.
